# Evaluation of the Sensory Quality and Shelf Life of a Bioactive Essence Rich in Monounsaturated Fatty Acids and Antioxidants, Obtained from Eco-Sustainable Iberian Ham

**DOI:** 10.3390/foods13223596

**Published:** 2024-11-11

**Authors:** Eva Bruna-García, Marta Miguel-Castro, Beatriz Isabel-Redondo

**Affiliations:** 1 Department of Bioactivity and Food Analysis, Institute of Food Science Research (CIAL, CSIC-UAM), 28049 Madrid, Spain; evabruna1@gmail.com; 2 Research and Development Department, Cárnicas Joselito S.A., 37156 Guijuelo, Spain; 3 Animal Production Department, Faculty of Veterinary, Complutense University of Madrid, 28040 Madrid, Spain

**Keywords:** quality, shelf life, healthy fats, Iberian ham

## Abstract

Food sustainability through traditional food production and the reuse of food by-products is one of the characteristics most valued by consumers. The production of Iberian ham is linked to the vaporization and sustainability of the dehesa and the conservation and maintenance of the rural environment, but there are some by-products that are not destined for direct consumption. In this context, previous studies have used trimmed fat to obtain a bioactive essence rich in antioxidants and monounsaturated fatty acids. Furthermore, it is important to keep in mind that the consumer’s decision is influenced by the nutritional/health and sensory characteristics of the product and its shelf life. The objective of the present study was to evaluate consumer acceptance and/or preference of different essences obtained from the trimmed fat of sliced Iberian ham and to determine the microbiological and physicochemical stability of the selected sustainable essence over time. The results showed that this essence is generally accepted by consumers and is microbiologically stable over time.

## 1. Introduction

Food sustainability is one of the characteristics most valued by today’s consumers, being at the forefront of research in agriculture and nutritional public health [1,2]. There are few foods in the world that come from privileged, respectful natural environments and whose nutritional richness derives from regenerative grazing systems. This is the case for acorn-fed Iberian ham from acorns, as the feeding regime of Iberian pigs is based on the use of resources from this pasture, feeding on acorns and grasses in an extensive production system, called “montanera” [3,4,5]. This traditional production system is closely linked to the valorization and sustainability of the dehesa and the conservation and maintenance of the rural environment, and plays an essential role in the survival of this ecosystem [6]. In addition, this valuable product has a great reputation among consumers, being one of the most emblematic products of Spanish gastronomy, with a high nutritional and sensory quality [5,7,8]. The yield of Iberian hams is high; however, there are some parts that are not intended for direct consumption [9,10]. In the past, the fat part of this Iberian ham was used to elaborate traditional recipes, but recently a bioactive essence has been obtained from the trimmed fat of sliced Iberian ham using a novel extraction method under vacuum conditions [10]. In quantitative terms, the reuse of this ham fat to obtain the bioactive essence means that for each sliced ham, 800 g of high-quality fat is recovered. Furthermore, it has been found that this essence has a high organoleptic quality, a high content of monounsaturated fatty acids (MUFAs), especially 56–58% oleic acid, antioxidant properties [10], and has been shown to act as a protective agent by counteracting the cellular effects of an oxidative insult, using Saccharomyces cerevisiae as a model organism [11]. In this context, antioxidants in food are currently being studied as they may prevent and/or protect against various diseases related to oxidative stress [10,12,13].

In addition to environmental concerns, the consumer’s decision to purchase a product is determined (with these characteristics being equally important) by the nutritional/healthy and sensory characteristics of the product and its shelf life [7]. In this context, one of the most widely used tests to check the acceptance of a product by consumers is through a hedonic test, which indicates how much a product is liked (sensory acceptance) or disliked (sensory rejection), followed by a preference test that indicates which samples of those evaluated are preferred by the consumer [11]. On the other hand, it is known that each product has its own shelf life [12,13] and must be established following current legislation. The objective of the present study was to evaluate consumer acceptance and/or preference of different essences obtained from the trimmed fat of sliced Iberian ham and to determine the microbiological and physicochemical stability of the selected sustainable essence over time.

## 2. Materials and Methods

### 2.1. Raw Material: Essences Derived from Cured Iberian Ham Fat

The fat used for this study was supplied by the company Cárnicas Joselito S.A. (Guijuelo, Spain) and was obtained during the slicing of Joselito hams. Joselito ham is a cured ham of the Iberian breed and is cured for a minimum of 36 months. The fat samples were received vacuum-packed in individual packages of approximately 2 kg and stored refrigerated (2 to 5 °C) until use, for a maximum of 20 days. For their use, the individual packages were opened, guaranteeing hygienic measures and avoiding possible cross-contamination. To obtain the essences, the samples were cut and prepared and the extraction process was carried out under vacuum conditions using a rotary evaporator as recently described at a temperature between 30 and 80 °C [10].

### 2.2. Hedonic and Preference Test/Analysis

The consumer acceptance study was conducted by means of an acceptability study using a hedonic test and a preference test [11,14] for the essences obtained in rotavapor at temperatures of 30, 60, and 80 °C as described by Bruna-García et al., (2022).

For each of the essences, the average of the olfactory, visual, and taste evaluation results was calculated, the percentage (%) of the sample preference was obtained, and an analysis was carried out to discriminate the results by age and sex/gender and to check the preference and acceptance in each population group. In addition, questions were included to obtain information from the consumer on how they would consume the product and whether they would make any modifications to it.

#### 2.2.1. Recruitment of Participants: Inclusion and Exclusion Criteria

The participants were chosen from different Spanish cities and with the following inclusion criteria: men and women, age range between 18 and 70 years, no allergy to ham and/or its fat, and preferably they liked this product.

#### 2.2.2. Preparation of Questionnaires

After the recruitment of the study participants, the necessary document for the sensory study was prepared. The document included basic instructions on how to prepare the sample and perform the subsequent analysis. In addition, a questionnaire was completed with questions relating to age, gender, occupation, allergies and consumption of medication, loss of taste and smell, and whether or not Iberian ham was frequently consumed. Finally, the document contained a questionnaire relating to the hedonic test and a survey oriented to their preferences in the purchase and consumption of similar products [11,14].

#### 2.2.3. Sample Preparation

All participants in this sensory study evaluated three essences selected from the essences obtained according to the methodology recently described by Bruna-García et al., (2022) [10]. Essences extracted from Joselito ham fat from the slicing process were evaluated under vacuum conditions (100 mbar) without solvents at temperatures of 30 °C, 60 °C, and 80 °C [10]. They were packaged under aseptic conditions to avoid microbiological contamination in 25 mL glass containers. Samples were then coded to avoid any bias in the evaluation by the participants, randomly assigning the code C01 to the sample extracted at 80 °C, B49 to the sample extracted at 60 °C, and A28 to the sample extracted at 30 °C. All samples were stored at −20 °C until use.

#### 2.2.4. Preparation of Shipments and Home Delivery

This study was carried out between November 2020 and November 2021. Taking into account the mobility and distance restrictions caused by the COVID-19 pandemic at the time of the study, it was decided to send the samples to each participant’s home. The shipment was carried using dry ice to ensure adequate transport conditions. The instructions for use and the questionnaire for the study were included.

### 2.3. Stability and Shelf Life Evaluation

#### 2.3.1. Sample Extraction and Preparation

For the stability and shelf life study, the essence extracted at 60 °C was used as described by Bruna-García et al. (2022) with modifications to ensure aseptic conditions and to obtain the final results of the analysis [10]. For this purpose, before extraction, all the material to be used was sterilized and a final amount of 3000 mL of sample was extracted. The sample was fractionated in duplicate in a horizontal laminar flow biosafety hood (Telstar Mini-H, Azbil Telstar, S.L.U., Terrassa, Barcelona, Spain) in airtight containers and stored under refrigeration (from 2 to 4 °C) and freezing (from −18 to −22 °C) conditions, under different conservation periods and under regulated conditions (control of the airtightness of the samples and adequacy of the temperature within the established margins). The different samples obtained in duplicate and stored were coded according to Table 1.

#### 2.3.2. Microbiological Analyses

According to the regulations, in this study it was decided to analyze the following reference microorganisms: aerobic and anaerobic mesophiles, spores of aerobic and anaerobic microorganisms [15], molds and yeasts [16], *Salmonella* [17], *Escherichia coli* (Brilliance™ *E. coli*), and *Listeria monocytogenes* [18]. When the analyses detected a quantity higher than the amount laid down in Regulation (EC) Nº 1441/2007 in a product, the essence was considered to have reached the end of its shelf life [19]. The maximum values laid down in the Regulation are shown in Table 2.

The determinations of the microbiological parameters analyzed were performed in duplicate. The results were expressed in colony-forming units/gram (cfu/g) and the results from *Listeria* monocytogenes and *Salmonella* spp. were reflected as the presence in 25 g of the sample.

### 2.4. Physicochemical Analyses

An analysis of the physical and chemical properties of samples under refrigeration and freezing conditions over time was also carried out. The samples under study and their coding are given in Table 1.

#### 2.4.1. Characterization of the Fatty Acid Composition

The fatty acid analysis of the different essences was performed according to the methodology described by Isabel et al., 2014, based on the extraction and acid methylation of fatty acid methyl esters with hexane, KOH, and methanol at temperatures of 65–70 °C, for subsequent injection and determination by gas chromatography with a flame ionization detector [20]. Fatty acids were identified by gas chromatography using a Hewlett Packard 6890 gas chromatograph (Hewlett-Packard Company (HP), Palo Alto, CA, USA), and a 30 m × 0.32 mm × 0.25 μm cross-linked polyethylene glycol capillary column was used (HP-INNOWAX, Agilent Technologies, St. Clara, CA, USA). A temperature program of 170 to 245 °C was used, and the injector and detector were kept at 250 °C. The flow rate of the carrier gas (helium) was 2 mL/min. Standard standards (Sigma, Alcobendas, Madrid, Spain) were used to identify each fatty acid. All samples were determined in triplicate and the results were expressed as percentages (%).

#### 2.4.2. Determination of the Degree of Acidity and Peroxide Index

To determine the degree of acidity and the peroxide index of the different samples, the official European analysis methods were used [21]. The two determinations were carried out in triplicate and the results of the degree of acidity are expressed in percentage of oleic acid (% Oleic Acid) and the peroxide index expressed in milliequivalents of active oxygen per kg of sample (meq O_2_/kg sample).

#### 2.4.3. pH Determination

The pH of the samples was determined with a digital pH meter (Hach Sension+ PH3, Vizcaya, Spain). Fifteen milliliters of each sample was immersed in the electrode and the pH was measured. This determination was carried out in triplicate and the results were expressed as pH units.

### 2.5. Statistical Analysis

The results were expressed as mean ± standard deviation (SD). Data were analyzed using Excel (Microsoft Office, Washington, WA, USA) and Graph-Pad Prism 10.00 statistical software for Windows (Graph-Pad Software, San Diego, CA, USA). One-way ANOVA was used as a statistical test, as appropriate, where a *p* value < 0.05 indicates that there were significant differences between two values.

## 3. Results and Discussion

### 3.1. Hedonic and Preference Test

**Figure 1:**
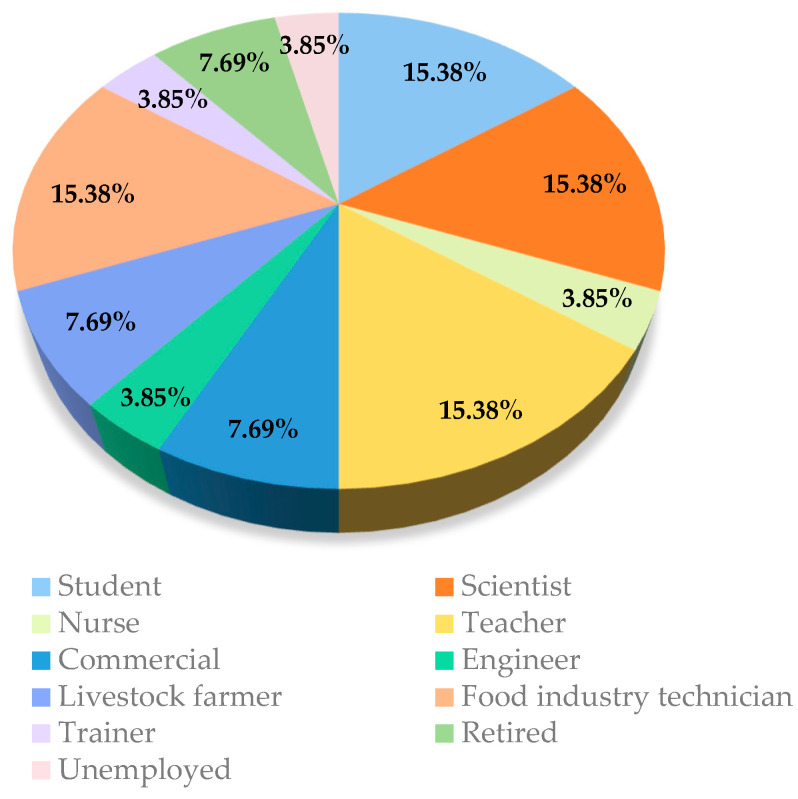
Percentages of occupations of study participants.

For the sensory evaluation of consumers, a five-point hedonic test and a consumer preference test were performed. These tests are a crucial tool in sensory evaluation, allowing the consumer’s liking and acceptability of a food product to be assessed [22,23]. Results were obtained from a total of 26 participants out of the 30 to whom samples were sent, 4 of whom ultimately decided not to participate in the study. Information on the final consumers who participated in the study is provided in the following Figures. The population that participated in the study had different professions and trades (scientists, farmers and ranchers, educators, or nurses, among others) (Figure 1).

**Figure 2:**
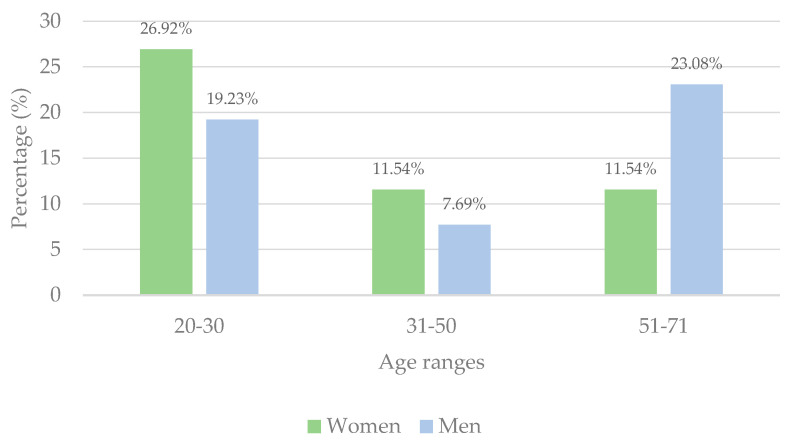
Percentages of age and gender of study participants.

As for the sex of the participants, of the total number of participants, 50% were women and 50% men, and if we divide them by age, we can see that the highest percentage of women were in the age range of 20–30 years (26.92%), while the majority of men who participated in the study were aged between 51 and 70 years (23.08%) (Figure 2). In addition, the participants were asked if they were regular consumers of Iberian ham, and 80.77% answered yes.

#### 3.1.1. Visual, Olfactory, and Taste Assessment

**Figure 3:**
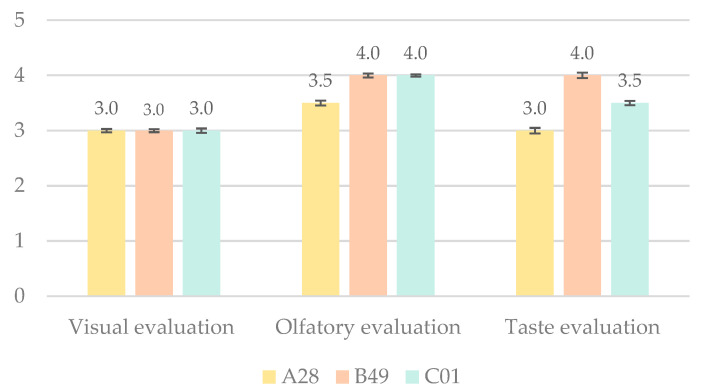
Results of the visual, olfactory, and taste sensory evaluation of the three samples under study.

The overall visual, olfactory, and gustatory evaluation was carried out with a five-point hedonic scale, and all the results were averaged with the mean of all of them. Appearance is the first characteristic perceived by the human senses and plays an important role in the identification and final selection of food [24]. Several studies have conducted sensory evaluation tests of dry-cured hams, focusing mainly on chemical and sensory modifications after storage, and the role of fat content in consumer acceptability, but by-products of Iberian ham have not been sensorially evaluated [25,26]. In terms of the scores obtained in the taste evaluation, sample B49 obtained the highest score (Figure 3). Smell and taste are sensory phenomena used to designate the sensations of smell, taste, and mouthfeel. Flavoring substances are aromatic compounds conceived by the combination of taste and smell and perceived by the mouth and nose and are very important for the final consumer [24].

**Figure 4:**
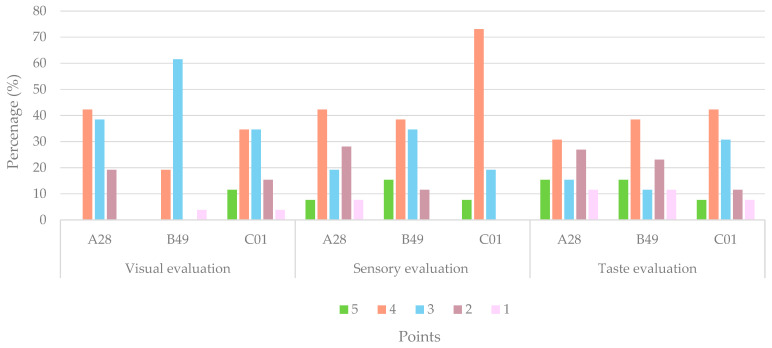
Percentage (%) scores of participants according to the points assigned to each sample.

Regarding visual evaluation, the highest score (5 points) was only given by 11.54% of the participants for sample C01, while samples A28 and B49 were not given scores of 5 points. Another aspect to highlight in the visual evaluation is the 42.31% of the participants who gave a score of 4 points to sample A28. Regarding the olfactory evaluation, the sample that obtained the highest score of 5 was sample B49, with 15.38% of the participants providing this rating, and, in addition, no participant rated it with the lowest possible score (1 point). On the other hand, sample C01 scored a much higher score of 4, which was given by 73.08% of the participants. Finally, with regard to the taste evaluation, the sample preferred by the participants was B49, with 15.38% rating it with 5 points and 38.46% with 4 points (Figure 4).

**Figure 5:**
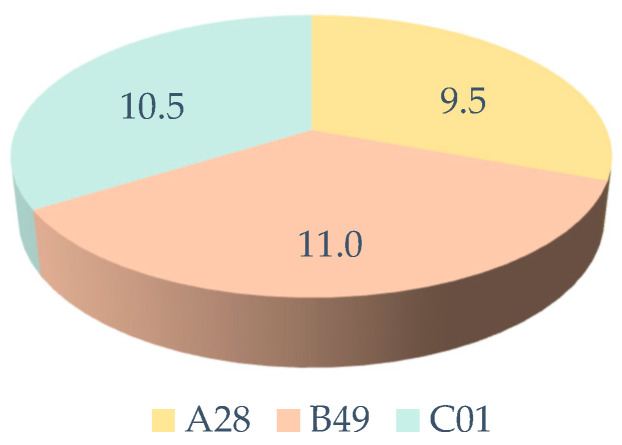
Total score (sum of the points obtained in the visual, olfactory, and taste evaluation).

The sum of these sensory characteristics is of great importance in the final consumer choice [27]. If we analyze the total results of the sum of the three evaluations (visual, olfactory, and taste), we can see that the sample with the highest total score was B49, followed by C01 and, finally, A28 (Figure 5). Color and texture are some of the most important characteristics in the consumer’s choice of meat products, so they can also be important in products derived from Iberian ham [27].

#### 3.1.2. Differences According to Age Group and Sex/Gender

It has been reported that sensory evaluation is strongly influenced by age and gender [24]. Differences in the preference of the samples under study were also analyzed according to the established age ranges (20–30, 31–50, 51–70 years). In the first group, the youngest population group, no differences in sample preference were observed, as all samples obtained the same percentage (33.3%). In the second population group (31–50 years), the least liked sample was C01, being voted for by 20% of the participants. Finally, in the last population group (51–70 years), sample C01 obtained the highest score, with 55.6% of the participants voting for it. On the other hand, when the preference samples by sex were analyzed, it was observed that men found B49 to be the best sample, with 53.85% of the votes, followed by C01 with 30.77% and finally A28 with 15.38% of the votes. In the case of the female participants, the results were different and were not as clear as in the case of the male participants, since samples A28 and C01 obtained an equal value (46.15%) and sample B49 obtained a very low value of 7.69%.

In summary, it can be indicated that significant differences were observed with the samples chosen between age groups and according to gender. We observed that the older age group preferred sample C01, which according to a previous study is associated with the sample that had a spicy and rancid aroma and taste [10]. If we distinguish by gender, men and women did not coincide in their choice of sample. For example, women are more sensitive than men to sweet and salty tastes and rate them as more pleasant [28,29].

#### 3.1.3. General Product Purchase and Acquisition Preferences and Information on Product Consumption and Modifications

Regarding purchase intention, 53.8% said they would buy the product, compared to 46.2% who said they would not buy it in the current market. On the other hand, focusing on the purchase preferences of the participants, it was found that 30.77% would buy sample A28, 30.77% would buy sample B49, and 38.46% would buy sample C10. According to a previous basic sensory study, all three samples under study had already been shown to have pleasant aromas [10]. These results refer to purchase preference, being only a higher acceptance of those characteristics in which the evaluator perceives that it differs from the other option(s) [30]. With respect to the consumption of the product, participants were asked to indicate how they would consume the product. In general terms, the most repeated responses, and taking into account that five of the participants chose not to respond and that some responded with more than one answer, were with bread, vegetables/salad, stews, and cheese/tofu/mayonnaise. These results are consistent with common uses of other vegetable oils/fats such as olive oil or peanut butter. On the other hand, 42.3% of the participants indicated that they would not make any modifications to the product. However, overall, the modifications that participants said they would make to the product were reducing the spiciness and bitterness in the mouth (26.9%) and reducing the fatty sensation and persistence in the mouth (30.8%).

### 3.2. Shelf Life Evaluation

During the shelf life of a food product, chemical, biochemical, physical and microbiological changes occur. Quality decline in most foods can be attributed to microbial growth being often correlated with sensory acceptability, thus being used as a marker, but also during storage physicochemical changes occur that influence quality deterioration, being attributable in most cases to oxidation reactions (Calligaris et al., 2016; Corradini et al., 2018) [15,16]. Shelf life can be defined as the period of time, under defined storage conditions, in which a food remains nutritionally, sensorially, and microbiologically acceptable for human consumption [12,13]. The European Union presents a regulation on microbiological criteria for foodstuffs [31], where microbiological criteria are defined as criteria that define the acceptability of a product, a batch of foodstuffs, or a process, based on the absence, presence, or number of microorganisms, and/or the amount of their toxins/metabolites, per unit mass, volume, surface, or batch [31].

#### 3.2.1. Microbiological Stability Study

Microbiological shelf life refers to the length of time during which a food product remains safe for consumption in terms of its microbiological quality. It is the period during which the microbial population within the product remains within acceptable limits, ensuring that the food is free of harmful microorganisms that may cause spoilage or pose health risks to consumers [32]. A microbiological stability study was carried out with the essence obtained at 60 °C according to the results obtained in previous studies [10,33].

The results obtained for the microbiological parameters analyzed according to Regulation N° 1441/2007 are shown in Table 3 and Table 4. No significant differences were observed in the duplicate samples and all microbiological parameters were within the established acceptable values. Therefore, the sample was microbiologically stable throughout the established times in freezing and refrigeration for all samples. The stability of the microbiological quality of dry-cured ham can be attributed to its low water activity [34]. If we look at time 0 corresponding to the extraction, an increase in mesophilic aerobes was observed in sample A with respect to the rest of the preserved samples.

Microbial growth is influenced by a number of factors intrinsic and extrinsic to the product that determine how fast microorganisms multiply and proliferate [32]. Foods rich in carbohydrates, proteins, and fats can provide an environment conducive to microbial growth. On the other hand, cold temperatures and a correct packaging method that decreases oxygen permeability and moisture slow microbial growth [32,35]. In the case of Iberian ham, we must not forget that during the salting phase, the microbial community formed colonizes the surface of the ham and is responsible for the formation of the aroma and flavor of this product [36,37]. Therefore, the microbiological quality of the fat used as raw material to obtain the essence is of vital importance. In this context, in this study, it was not observed that microbial growth was significantly decreased in the samples preserved by freezing with respect to the samples preserved by refrigeration, being very stable over time. If we compare the evaluated sample with olive oil, it was established that the shelf life of olive oil is 12–18 months, although it has been shown that when properly stored, it can withstand a second year of storage [38], so the results of the essence extracted at 60 °C and evaluated agree with those of olive oil.

#### 3.2.2. Characterization of the Fatty Acid Composition

The characterization of the fatty acid (FA) composition was carried out on the essences obtained at 60 °C and preserved under refrigeration and freezing over time. Table 5 and Table 6 show the results obtained for saturated (SFAs), monounsaturated (MUFAs) and polyunsaturated (PUFAs) fatty acids of the different samples obtained at 60 °C.

The results for the refrigerated essence obtained at 60 °C did not show significant differences when every fatty acid was analyzed individually; however, significant differences (*p* < 0.05) in the total SFAs, MUFAs, and PUFAs were observed (Table 5). The lowest total SFA values were obtained in the raw material (27.60 ± 0.02) and the highest were those obtained after eight months (31.60 ± 0.09). Total MUFAs at T0 were significantly higher than in the samples preserved over time under refrigeration. On the contrary, total PUFA composition fluctuated during the refrigerated storage period, with increases at 4 and 8 months. Table 6 shows the results obtained for the fatty acid composition of the essence obtained at 60 °C and kept frozen. No significant differences were observed when the fatty acid composition was analyzed individually, but significant differences (*p* < 0.05) in the concentrations of SFAs, MUFAs, and total PUFAs were observed. Again, the SFA results of the T0 were significantly lower (27.60 ± 0.02) and the highest were obtained in sample F-6M (34.37 ± 0.06), being significantly higher than the rest of the samples analyzed. Samples R-1M and R-4M did not show significant differences in their total SFA composition. The MUFAs at T0 (64.88 ± 0.02) were significantly higher than the rest of the samples analyzed, and in samples F-1M, F-4M and F-10M, no significant differences were observed. In the composition of PUFAs, significant differences were observed at T0, showing the highest value (7.53 ± 0).

In both types of essence preserved over time, as previously described by Bruna-García et al. (2022), a high concentration of MUFA was observed, highlighting the concentrations of oleic acid in all the samples under study [10]. It has been shown that one of the most characteristic effects of oleic acid is its antioxidant capacity, since it can directly regulate both the synthesis and the activities of antioxidant enzymes. This antioxidant capacity may be related to the antihypertensive effect attributed to the improvement of endothelial dysfunction. In addition, antioxidants reduce cholesterol absorption and decrease low-density lipoprotein (LDL) oxidation, which prevents atherosclerosis [39]. On the other hand, linoleic acid is the predominant PUFA in all samples and is associated with a lower incidence of cardiovascular diseases, metabolic syndrome, or type 2 diabetes, and α-linolenic acid has demonstrated protective effects against hypertension, contributing to balanced blood pressure [40,41].

#### 3.2.3. Determination of the Degree of Acidity and Peroxide Index

The degree of acidity and peroxide index of the essence samples were analyzed over time under refrigeration and freezing (Table 7 and Table 8). Table 7 shows the results obtained for the essence obtained at 60 °C kept under refrigeration over time. It is observed that both the degree of acidity and peroxides increased significantly over the storage time when compared to T0. A maximum was found after 6 months in the case of acidity and after 4 months for peroxides, values that decreased at later times but without reaching the T0 values.

With respect to the results of the essence obtained at 60 °C preserved by freezing over time (Table 8), it is observed that the degree of acidity presented a significantly higher value in the F-6M sample corresponding to 6 months of storage and the peroxides of the essence increased significantly over the storage time compared to T0. The peroxide index increased significantly throughout the frozen storage time, reaching the maximum value at 15 months (6.52 ± 0.11), at the end of the study.

The degree of acidity is a measure of the amount of free fatty acids in the oil [10,42] and the peroxide index is an important parameter that defines the quality of oils in terms of primary oxidative products [43]. Fats break down into different oxidation by-products over time, and when lipid oxidation takes place in foods during storage, it leads to the formation of undesirable flavors and/or colors, making foods less acceptable or totally unacceptable [38]. Peroxides slowly accumulate over time, contributing to oil rancidity and giving rise to undesirable flavors and odors, which can alter its shelf life [10]. Alvarruiz et al. observed that the values of acidity and peroxides obtained from olive oil stored at 5 °C for 3 years showed fluctuations over time and none of them followed a linear pattern [43], similar to what happened with the results obtained in our study. Oxidation in food is a complex set of reactions involving molecules belonging to the lipid family and oxygen, leading to the formation of a series of radical and highly reactive species. Although lipid oxidation occurs and can cause food spoilage during storage, it should be noted that the only mandatory indication relevant to the development of oxidation in food is the one for olive oils [12,31].

#### 3.2.4. pH Determination

The determination of the pH of foods over time is also important to determine their stability, and in addition, the pH value is one of the most important properties related to the quality of dry-cured ham [44].

The pH of the refrigerated and frozen samples was determined over time and compared with that of the raw material. The pH values of the refrigerated samples were between 5.20 ± 0.14 and 6.03 ± 0.18, showing significant differences between the results. The results of the samples preserved by freezing did not show significant differences, obtaining values between 5.20 ± 0.14 and 5.8 ± 0.33. In the refrigerated and frozen samples, it was observed that the pH generally increased with storage time, with the results obtained coinciding with the decrease in acidity after 8 months in refrigeration and 15 months in freezing. Piras et al. observed that for cured ham packaged and stored for different days and at different temperatures, the pH values ranged between 5.8 and 6.3 and the temperature did not influence pH values during storage [33].

## 4. Conclusions

Today, foods that come from planet-friendly environments and whose nutritional richness derives from regenerative grazing systems and the philosophy of the circular economy and the use of food by-products for the creation of bioactive foods that provide health benefits to consumers are in short supply. To achieve these new sustainable products, food design is necessary and, in addition, the shelf life of a product is as important as the opinion that the consumer has of it, so that the designed food can reach the market. For the essences obtained in a rotary evaporator at 30, 60, and 80 °C that were evaluated by consumers, a general acceptance of all of them was observed, with a general preference for the sample obtained at 60 °C. Specifying by sex and age, we observed discrepancy in the results. Furthermore, it has been shown that the essence obtained from ham fat under vacuum conditions at a temperature of 60 °C is microbiologically stable over time when stored under refrigeration (1 week–8 months) and freezing (1 week–15 months) conditions. On the other hand, it was also observed that, using both conservation methods, a high concentration of AGMI was obtained in all the essences under study, with the oleic acid concentrations being particularly noteworthy. The results obtained for the peroxide index, the degree of acidity, and the pH showed very similar values over time both in refrigerated and frozen storage and did not show any notable alterations. Thus, it was possible to obtain a sustainable essence that brings together the main characteristics most appreciated by consumers: nutrition, flavor, sustainability, and shelf life.

## Figures and Tables

**Table 1:** Coding of the samples obtained.

Analysis Time	Refrigeration (2 to 4 °C )	Freezing (−18 to −22 °C )
Time 0	T0	
1 week	R-1W	F-1W
1 month	R-1M	F-1M
4 months	R-4M	F-4M
6 months	R-6M	F-6M
8 months	R-8M	F-8M
10 months	-	F-10M
15 months	-	F-15M

**Table 2:** Maximum permitted microbiological values.

Microorganism Tested	Detection Limit
Aerobic mesophiles	10^3^ cfu/gr
Anaerobic mesophiles	10^2^ cfu/gr
Anaerobic spores	10^2^ cfu/gr
Aerobic spores	10^2^ cfu/gr
Fungi and yeasts	10^2^ cfu/g
*Escherichia coli*	10^2^ cfu/gr
*Salmonella* spp.	Absence in 25 g
*Listeria monocytogenes*	Absence in 25 g

**Table 3:** Microbiological results obtained from refrigerated samples and at time 0 of analysis.

Microorganism	Sample											
	T0		R-1S		R-1M		R-4M		R-6M		R-8M	
	A *	B *	A *	B *	A *	B *	A *	B *	A *	B *	A *	B *
Aerobic mesophiles	5 × 10^1^	1 × 10^1^	5 × 10^1^	<10^1^	1 × 10^1^	<10^1^	<10^1^	<10^1^	<10^1^	<10^1^	<10^1^	1.1 × 10^2^
Anaerobic mesophiles	<10^1^	<10^1^	<10^1^	<10^1^	<10^1^	<10^1^	<10^1^	<10^1^	<10^1^	<10^1^	<10^1^	<10^1^
Anaerobic spores	<10^1^	1 × 10^1^	<10^1^	<10^1^	1 × 10^1^	<10^1^	1 × 10^1^	<10^1^	1 × 10^1^	<10^1^	<10^1^	<10^1^
Aerobic spores	<10^1^	<10^1^	<10^1^	<10^1^	<10^1^	<10^1^	<10^1^	<10^1^	<10^1^	<10^1^	<10^1^	<10^1^
Fungi and yeasts	<10^2^	<10^2^	<10^2^	<10^2^	<10^2^	<10^2^	<10^2^	<10^2^	<10^2^	<10^2^	<10^2^	<10^2^
*Escherichia coli*	<10^2^	<10^2^	<10^2^	<10^2^	<10^2^	<10^2^	<10^2^	<10^2^	<10^2^	<10^2^	<10^2^	<10^2^
*Salmonella* spp.	Abs. 25gr **	Abs. 25gr **	Abs. 25gr **	Abs. 25gr **	Abs. 25gr **	Abs. 25gr **	Abs. 25gr **	Abs. 25gr **	Abs. 25gr **	Abs. 25gr **	Abs. 25gr **	Abs. 25gr **
*Listeria monocytogenes*	Abs. 25gr **	Abs. 25gr **	Abs. 25gr**	Abs. 25gr **	Abs. 25gr **	Abs. 25gr **	Abs. 25gr **	Abs. 25gr **	Abs. 25gr **	Abs. 25gr **	Abs. 25gr **	Abs. 25gr **

* A: indicates first duplicate sample taken; B: indicates second duplicate sample taken. ** Abs. 25gr: indicates absence in 25 g of sample.

**Table 4:** Microbiological results obtained from freezing samples and at time 0 of analysis.

Microorganism	Sample															
	T0		F-1S		F-1M		F-4M		F-6M		F-8M		F-10M		F-15M	
	A *	B *	A *	B *	A *	B *	A *	B *	A *	B *	A *	B *	A *	B *	A *	B *
Aerobic mesophiles	5 × 10^1^	1 × 10^1^	<10^1^	<10^1^	<10^1^	<10^1^	<10^1^	<10^1^	1 × 10^1^	<10^1^	<10^1^	<10^1^	1 × 10^2^	1.1 × 10^2^	<10^1^	<10^1^
Anaerobic mesophiles	<10^1^	<10^1^	<10^1^	<10^1^	<10^1^	<10^1^	<10^1^	<10^1^	3 × 10^1^	<10^1^	<10^1^	<10^1^	<10^1^	<10^1^	<10^1^	<10^1^
Anaerobic spores	<10^1^	1 × 10^1^	1 × 10^1^	<10^1^	1 × 10^1^	<10^1^	<10^1^	<10^1^	<10^1^	<10^1^	1 × 10^1^	<10^1^	<10^1^	<10^1^	1 × 10^1^	<10^1^
Aerobic spores	<10^1^	<10^1^	<10^1^	<10^1^	<10^1^	<10^1^	<10^1^	<10^1^	<10^1^	<10^1^	<10^1^	<10^1^	<10^1^	<10^1^	<10^1^	<10^1^
Fungi and yeasts	<10^2^	<10^2^	<10^2^	<10^2^	<10^2^	<10^2^	<10^2^	<10^2^	<10^2^	<10^2^	<10^2^	<10^2^	<10^2^	<10^2^	<10^2^	<10^2^
*Escherichia coli*	<10^2^	<10^2^	<10^2^	<10^2^	<10^2^	<10^2^	<10^2^	<10^2^	<10^2^	<10^2^	<10^2^	<10^2^	<10^2^	<10^2^	<10^2^	<10^2^
*Salmonella* spp.	Abs. 25gr **	Abs. 25gr **	Abs. 25gr **	Abs. 25gr **	Abs. 25gr **	Abs. 25gr **	Abs. 25gr **	Abs. 25gr **	Abs. 25gr **	Abs. 25gr **	Abs. 25gr **	Abs. 25gr **	Abs. 25gr **	Abs. 25gr **	Abs. 25gr **	Abs. 25gr **
*Listeria monocytogenes*	Abs. 25gr **	Abs. 25gr **	Abs. 25gr **	Abs. 25gr **	Abs. 25gr **	Abs. 25gr **	Abs. 25gr **	Abs. 25gr **	Abs. 25gr **	Abs. 25gr **	Abs. 25gr **	Abs. 25gr **	Abs. 25gr **	Abs. 25gr **	Abs. 25gr **	Abs. 25gr **

* A: indicates first duplicate sample taken; B: indicates second duplicate sample taken. ** Abs. 25gr: indicates absence in 25 g of sample.

**Table 5:** Fatty acid composition of essences preserved under refrigeration.

Fatty Acid		Sample					
		T0	R-1W	R-1M	R-4M	R-6M	R-8M
**SFAs (%)**	Lauric	0.05 ± 0	0.03 ± 0	0.05 ± 0	0.06 ± 0.01	0.06 ± 0	0.68 ± 0
	Myristic	1.05 ± 0.03	1.16 ± 0.02	1.10 ± 0.02	1.16 ± 0.02	1.15 ± 0.03	1.16 ± 0
	Palmitic	19.62 ± 0.02	21.44 ± 0.12	20.01 ± 0.01	20.27 ± 0.11	20.25 ± 0.16	21.52 ± 0.03
	Margaric	0.19 ± 0	0.25 ± 0.12	0.19 ± 0	0.24 ± 0	0.26 ± 0.1	0.25 ± 0
	Stearic	6.52 ± 0.1	8.27 ± 0.08	9.43 ± 0.05	8.99 ± 0.05	9.37 ± 0.08	8.44 ± 0.06
	Arachidic	0.12 ± 0.01	0.13 ± 0.01	0.16 ± 0	0.15 ± 0.01	0.17 ± 0.01	0.17 ± 0
	**Total SFAs (%)**	27.60 ± 0.02 ^a^	31.23 ± 0.16 ^c^	31.00 ± 0.01 ^b^	30.81 ± 0.07 ^b^	31.32 ± 0.14 ^c^	31.60 ± 0.09 ^d^
**MUFAs (%)**	Palmitoleic	2.41 ± 0	2.20 ± 0.03	2.08 ± 0.01	2.26 ± 0.01	2.08 ± 0.01	2.20 ± 0.02
	Cis-10-heptadecenoic	0.25 ± 0.01	0.29 ± 0.01	0.27 ± 0	0.28 ± 0.01	0.28 ± 0.01	0.29 ± 0.01
	Oleic	60.95 ± 0.02	57.63 ± 0.15	57.50 ± 0.11	58 ± 0.09	57.45 ± 0.11	57.35 ± 0.09
	Eicosanoid	1.26 ± 0.02	1.35 ± 0.03	1.36 ± 0	1.26 ± 0	1.26 ± 0.04	1.36 ± 0.03
	**Total MUFAs (%)**	64.88 ± 0.02 ^d^	61.50 ± 0.21 ^b^	61.21 ± 0.01 ^a^	61.82 ± 0.07 ^c^	61.00 ± 0.13 ^a^	61.20 ± 0.08 ^a^
**PUFAs (%)**	Linoleic	7.05 ± 0.01	6.62 ± 0.01	7.18 ± 0.01	6.81 ± 0	7.10 ± 0.01	6.60 ± 0.02
	α-linolenic	0.48 ± 0.01	0.64 ± 0.01	0.61 ± 0.01	0.57 ± 0.01	0.60 ± 0.01	0.61 ± 0.02
	**Total PUFAs (%)**	7.53 ± 0 ^b,c^	7.30 ± 0.01 ^a^	7.79 ± 0.01 ^d^	7.37 ± 0.01 ^a,b^	7.67 ± 0.05 ^c,d^	7.21 ± 0.01 ^a^

Results expressed as mean ± SD (n = 3). Different letters (a, b, c or d) in the same row show significant differences (*p* < 0.05). SFAs: saturated fatty acids; MUFAs: monounsaturated fatty acids; PUFAs: polyunsaturated fatty acids.

**Table 6:** Fatty acid composition of the essences preserved in freezing.

Fatty Acid (%)		Sample							
		T0	F-1W	F-1M	F-4M	F-6M	F-8M	F-10M	F-15M
**SFAs (%)**	Lauric	0.05 ± 0	0.06 ± 0	0.05 ± 0.01	0.06 ± 0	0.06 ± 0	0.06 ± 0	0.05 ± 0	0.05 ± 0
	Myristic	1.05 ± 0.03	1.12 ± 0.02	1.09 ± 0.02	1.20 ± 0.01	1.17 ± 0.02	1.15 ± 0.01	1.05 ± 0	1.03 ± 0.02
	Palmitic	19.62 ± 0.02	21.23 ± 0.24	21.45 ± 0.12	20.71 ± 0.11	21.27 ± 0.29	20.88 ± 0.34	20.86 ± 0.14	19.79 ± 0.08
	Margaric	0.19 ± 0	0.25 ± 0.01	0.24 ± 0.04	0.23 ± 0	0.24 ± 0.02	0.23 ± 0.01	0.23 ± 0	0.23 ± 0.01
	Stearic	6.52 ± 0.1	8.32 ± 0.05	9.34 ± 0.04	8.87 ± 0.05	11.42 ± 0.04	10.16 ± 0	8.81 ± 0	8.82 ± 0.03
	Arachidic	0.12 ± 0.01	0.15 ± 0.01	0.16 ± 0.01	0.16 ± 0.01	0.20 ± 0.01	0.16 ± 0	0.15 ± 0.01	0.16 ± 0.01
	**Total SFAs (%)**	27.60 ± 0.02 ^a^	31.12 ± 0.10 ^c^	32.33 ± 0.10 ^e^	31.22 ± 0.07 ^c,d^	34.37 ± 0.06 ^g^	32.64 ± 0.05 ^f^	31.37 ± 0.15 ^d^	30.08 ± 0.06 ^b^
**MUFAs (%)**	Palmitoleic	2.41 ± 0	2.19 ± 0.03	2.05 ± 0.01	2.31 ± 0.03	1.95 ± 0.02	2.07 ± 0.01	1.97 ± 0.01	1.98 ± 0.02
	Cis-10-heptadecenoic	0.25 ± 0.01	0.28 ± 0.01	0.26 ± 0.01	0.26 ± 0.01	0.22 ± 0.01	0.25 ± 0.01	0.22 ± 0.01	0.25 ± 0.01
	Oleic	60.95 ± 0.02	57.72 ± 0.11	56.91 ± 0.09	57.60 ± 0.09	55.12 ± 0.09	56.54 ± 0.28	58.46 ± 0.33	58.99 ± 0.62
	Eicosanoid	1.26 ± 0.02	1.38 ± 0.02	1.36 ± 0.03	1.37 ± 0.01	1.32 ± 0.03	1.28 ± 0.02	1.22 ± 0.02	1.25 ± 0.03
	**Total MUFAs (%)**	64.88 ± 0.02 ^f^	61.56 ± 0.09 ^d^	60.58 ± 0.10 ^c^	61.55 ± 0.06 ^d^	58.61 ± 0.08 ^a^	60.14 ± 0.03 ^b^	61.71 ± 0.08 ^d^	62.47 ± 0.06 ^e^
**PUFAs (%)**	Linoleic	7.05 ± 0.01	6.67 ± 0.01	6.51 ± 0.02	6.71 ± 0.01	6.53 ± 0.01	6.68 ± 0.01	6.48 ± 0.03	6.92 ± 0.01
	α-linolenic	0.48 ± 0.01	0.65 ± 0.01	0.58 ± 0.02	0.52 ± 0.01	0.49 ± 0.02	0.54 ± 0.02	0.49 ± 0.01	0.54 ± 0.16
	**Total PUFAs (%)**	7.53 ± 0 ^g^	7.32 ± 0.02 ^d,e,f^	7.09 ± 0.01 ^b,c^	7.24 ± 0.01 ^c,e^	7.02 ± 0.03 ^a,b^	7.22 ± 0.02 ^c,d^	6.91 ± 0.07 ^a^	7.46 ± 0.01 ^f,g^

Results expressed as mean ± SD (n = 3). Different letters (a, b, c, d, e, f, or g) in the same row show significant differences (*p* < 0.05). SFAs: saturated fatty acids; MUFAs: monounsaturated fatty acids; PUFAs: polyunsaturated fatty acids.

**Table 7:** Results of the degree of acidity and peroxides obtained for the essence obtained at 60 °C kept under refrigeration over time.

Sample	Degree of Acidity (% Oleic Acid)	Peroxide Value (Meq O_2_/kg Fat)
T0	24.02 ± 0.10 ^a^	2.78 ± 0.16 ^a^
R-1S	24.94 ± 0.03 ^a,c^	4.10 ± 0.09 ^b^
R-1M	25.81 ± 0.12 ^b,c^	5.03 ± 0.11^c^
R-4M	25.16 ± 0.38 ^a,c^	5.82 ± 0.30 ^d^
R-6M	27.88 ± 0.19 ^d^	4.42 ± 0.14 ^b,c^
R-8M	24.92 ± 0.67 ^a,b^	4.58 ± 0.08 ^b,c^

Mean ± standard deviation. Different letters (a, b, c, or d) in each column show significant differences (*p* < 0.05).

**Table 8:** Results obtained for the essence obtained at 60 °C kept under freezing conditions over time.

Sample	Degree of Acidity (% Oleic Acid)	Peroxide Value (Meq O_2_/kg Fat)
T0	24.02 ± 0.10 ^a^	2.78 ± 0.16 ^a^
F-1S	24.99 ± 0.01^a,c^	4.50 ± 0.33 ^b^
F-1M	25.75 ± 0.27 ^b,c^	4.40 ± 0.66 ^b^
F-4M	26.99 ± 0.77 ^d^	3.86 ± 0.13 ^b^
F-6M	30.70 ± 0.4 ^e^	4.38 ± 0.8 ^b^
F-8M	24.46 ± 0.62 ^a^	5.56 ± 0.17 ^c^
F-10M	26.26 ± 0.06 ^d^	5.27 ± 0.16 ^c^
F-15M	24.66 ± 0.05 ^a,b^	6.52 ± 0.11^d^

Mean ± standard deviation. Different letters (a, b, c, d, or e) in each column show significant differences (*p* < 0.05).

## Data Availability

The original contributions presented in the study are included in the article, further inquiries can be directed to the corresponding authors.
